# Suicidal ideation in female individuals with fibromyalgia and comorbid obesity: prevalence and association with clinical, pain-related, and psychological factors

**DOI:** 10.1093/pm/pnad139

**Published:** 2023-10-16

**Authors:** Giorgia Varallo, Federica Scarpina, Tor Arnison, Emanuele Maria Giusti, Micheal Tenti, Giada Rapelli, Roberto Cattivelli, Giulia Landi, Eliana Tossani, Silvana Grandi, Christian Franceschini, Valentina Baldini, Giuseppe Plazzi, Paolo Capodaglio, Gianluca Castelnuovo

**Affiliations:** Department of Biomedical, Metabolic and Neural Sciences, University of Modena and Reggio Emilia, Modena 41125, Italy; Istituto Auxologico Italiano, IRCCS, U.O. di Neurologia e Neuroriabilitazione, Ospedale San Giuseppe, Piancavallo 28884, Italy; “Rita Levi Montalcini” Department of Neurosciences, University of Turin, Turin 10126, Italy; Clinical Epidemiology and Biostatistics, Faculty of Medicine and Health, School of medical Sciences, Örebro University, Örebro 70182, Sweden; EPIMED Research Center, Department of Medicine and Surgery, University of Insubria, Varese 21100, Italy; Institute for Research on Pain, ISAL Foundation, Rimini 47921, Italy; Department of Medicine and surgery, University of Parma, Parma 43125, Italy; Department of Psychology “Renzo Canestrari”, Alma Mater Studiorum, University of Bologna, Bologna 40126, Italy; Department of Psychology “Renzo Canestrari”, Alma Mater Studiorum, University of Bologna, Bologna 40126, Italy; Department of Psychology “Renzo Canestrari”, Alma Mater Studiorum, University of Bologna, Bologna 40126, Italy; Department of Psychology “Renzo Canestrari”, Alma Mater Studiorum, University of Bologna, Bologna 40126, Italy; Department of Medicine and surgery, University of Parma, Parma 43125, Italy; Department of Biomedical, Metabolic and Neural Sciences, University of Modena and Reggio Emilia, Modena 41125, Italy; Department of Biomedical and Neuromotor Sciences, University of Bologna, Bologna 40126, Italy; Department of Biomedical, Metabolic and Neural Sciences, University of Modena and Reggio Emilia, Modena 41125, Italy; IRCCS Istituto delle Scienze Neurologiche di Bologna, Bologna 40139, Italy; Laboratory of Biomechanics, Rehabilitation and Ergonomics, IRCCS, Istituto Auxologico Italiano, Verbania 28884, Italy; Department of Surgical Sciences, Physical Medicine and Rehabilitation, University of Torino, Turin 10124, Italy; Department of Psychology, Catholic University of the Sacred Heart of Milan, Milan 20123, Italy; IRCCS Istituto Auxologico Italiano, Psychology Research Laboratory, Ospedale San Giuseppe, Verbania 28884, Italy

**Keywords:** fibromyalgia, suicidal ideation, obesity, pain catastrophizing, depression, sleep quality

## Abstract

**Objective:**

Individuals with fibromyalgia report alarming levels of suicidal ideation, and comorbidity with other chronic health conditions such as obesity—a risk factor for suicidal ideation per se—could further complicate the clinical picture. The aim of this study is to determine, in a sample of women with fibromyalgia and comorbid obesity, the prevalence of suicidal ideation and to evaluate clinical, pain-related and psychological factors associated with suicidal ideation.

**Methods:**

In total, 156 female individuals with fibromyalgia and obesity were recruited and completed a series of self-report measures that assessed (i) the level of pain intensity, (ii) depressive symptomatology, (iii) sleep quality, and (iv) pain catastrophizing. Suicidal ideation was evaluated by item #9 of the Beck Depression Inventory. In addition, information regarding previous suicide attempts and current opioid use was collected.

**Results:**

3n sum, 7.8% of participants reported presence of suicidal ideation. According to the results of the multiple logistic regression, depressive symptomatology, sleep quality, and pain catastrophizing were associated with the presence of suicidal ideation.

**Discussion:**

The presence of suicidal ideation in our sample was significantly associated with depressive symptomatology, sleep quality, and pain catastrophizing. Our findings are the first to suggest a unique (ie, independent of depressive symptomatology, and sleep quality) association between pain catastrophizing and suicidal ideation in the context of fibromyalgia and comorbid obesity. In order to prevent and reduce suicidal ideation, these factors should be assessed and targeted in interventions for pain management. Future research should investigate the extent to which addressing depressive symptoms, sleep quality, and pain catastrophizing reduces suicidal ideation.

## Introduction

At some point in our lives, we inevitably encounter pain, often in the form of an acute experience that typically resolves. However, in certain cases, the pain persists beyond its usual course and transforms into a chronic condition. Nevertheless, chronic pain is more than a persistent and unpleasant sensory experience. Indeed, it exerts a profound impact on various domains of life.^1^ Living with chronic pain not only takes a toll on the body but also emotional well-being. Everyday activities such as self-care, work, and social interactions become arduous tasks, presenting constant challenges.^2^ Thus, it is not surprising that affected individuals experience poor physical, psychological, and social wellbeing to the point that chronic pain is ranked among the conditions associated with the lowest quality of life indices when compared to other chronic health conditions.^3^^,^^4^ The psychological burden of chronic pain is high, in fact, psychiatric comorbidities are prevalent and often co-occurring with up to 60% of chronic pain patients also presenting with depression.^5^^,^^6^

In addition to the severe impairment experienced by affected individuals in the physical, psychological, and social domains, evidence has suggested that chronic pain patients are at a heightened risk for suicidal behaviors.^7^ Notably, suicidal behaviors encompass not only suicide attempts and suicide per se but also suicidal thoughts, desires, and preoccupations (ie, suicidal ideation [SI]). The rates of SI and suicide attempts are 2–3 times higher in individuals with chronic pain compared to the general population.^7^ Considering that SI strongly predicts suicide attempts and death by suicide,^8^ and that more than 60% of suicide attempts occur within the first year after the onset of SI,^9^ it becomes imperative to closely monitor and address this aspect in order to prevent suicides.

The focus of our investigation is on fibromyalgia (FM), which is a chronic pain condition characterized not only by widespread pain and mood disorders, including depression, but also by accompanying symptoms such as severe fatigue, poor sleep quality, and cognitive impairment.^10^ It affects between 0.2% and 6.6% of the global population, with a higher prevalence rate among women (between 2.4% and 6.8%).^11^ It is regarded as one of the most challenging chronic pain conditions to manage.^12^ Living with FM necessitates constant adjustment across various aspects of life, from work obligations to parenting responsibilities and social participation. Individuals with FM may struggle to fulfill their familiar duties^13^ and have a high rate of absenteeism and decreased work productivity.^14^ They usually face social isolation, including rejection by family, friends, and health care providers.^15^ A recent systematic review have reported SI prevalence rates in people with FM ranging from 26.5 to 58.3%,^16^ a remarkably high occurrence when compared to the general population prevalence of 9.2%.^9^ FM patients frequently report feeling neglected by the healthcare system due to the condition’s uncertain etiology. As a consequence, they often obtain a definitive diagnosis several years after the onset of symptoms,^17^ while receiving inadequate or insufficient treatment. This aspect poses a particular concern, as SI is likely to be disregarded and left untreated when there are delays in diagnosis and the initiation of appropriate treatment. Treatment options for FM encompass a multimodal approach, that include evidence-based pharmacological and non-pharmacological interventions.^18^ However, in the last decade, a concerning trend has emerged, characterized by a notable rise in the prescription and utilization of opioid for FM.^19^ Opioid lack empirical evidence supporting their effectiveness in the treatment of FM, a fact consistently emphasized in clinical guidelines^20^ and their use introduces an array of additional hazards, including the potential exacerbation of suicidal behavior.^21–24^

FM is frequently associated with other health conditions posing a significant burden on individuals' quality of life. Among these comorbidities, obesity stands out as both a common occurrence (with a prevalence of 35.7%^25^) and an independent risk factor for SI. Notably, elevated body mass index (BMI) has been associated with major depression and SI in female individuals (but not male), highlighting the gender-specific influence.^26^ These patients face a double disease burden due to the impairment caused by two chronic and disabling conditions, with additional detrimental effects on their quality of life and wellbeing. Patients with FM and obesity reported higher levels of pain intensity,^27^^,^^28^ sleep disturbances,^29^ and depression^28^^,^^29^ compared to their normal and overweight counterparts. Hence, female patients with FM and obesity may represent an especially vulnerable population that has been inadequately explored in research.

While there is a growing body of research on chronic pain and SI, the focus on FM remains relatively limited. Considering the vast heterogeneity of chronic pain conditions in terms of clinical manifestations, impairment, and comorbidities, investigating condition-specific factors becomes imperative. Few preliminary evidence suggested that SI is associated with a number of factors in FM, with depression, poor sleep quality, and pain intensity emerging as key contributors.^30–32^ Despite the growing interest in the impact of psychological factors on FM, few studies have evaluated their impact on SI. A recent systematic review^16^ highlighted the paucity of research on suicidality and FM and suggested examining the role of pain catastrophizing. Indeed, pain catastrophizing is associated with SI in patients with chronic pain^33^^,^^34^ and even more importantly is modifiable through psychological interventions.^35^ Pain catastrophizing has garnered substantial attention as one of the most consistent predictors of pain intensity,^36^^,^^37^ physical function,^38–40^ and psychological health.^37^ It is defined as negative cognitive–affective response to anticipated or actual pain that involve 3 major components: Magnification (eg, “I’m afraid that something serious might happen”), rumination (“I cańt stop thinking about how much it hurts”), and helplessness (eg, “There is nothing I can do to reduce the intensity of my pain”).^41^^,^^42^ However, the available evidence is limited and predominantly pertains to mixed chronic pain populations, with a dearth of data specifically exploring SI among female patients with FM, particularly those with comorbid obesity. Identification of psychological risk factors modifiable via psychological interventions,^35^ such as pain catastrophizing, could provide intervention targets for preventing the progression of SI toward more severe suicidal behaviors.

Consequently, our objectives were 2-fold: (*i)* to evaluate the prevalence of SI and (*ii)* to explore the relationship between SI and clinical (ie, body mass index, previous suicide attempt, current opioid use, sleep quality), pain-related (ie, pain intensity, pain duration) and psychological factors (ie, depressive symptomatology and pain catastrophizing) in a sample of female patients with FM and obesity.

## Methods

### Study design and procedure

A cross-sectional study was undertaken as part of a broader research initiative that received approval from the ethics committee of Istituto Auxologico Italiano IRCCS (code: 2021_05_18_14). The data collection phase spanned from June 2021 to September 2022 at the Rehabilitation Unit of Istituto Auxologico Italiano IRCCS, situated in Piancavallo, Italy, which provides specialized care for both musculoskeletal rehabilitation and weight reduction.

Participants were included according to the following criteria: (a) age > 18 years, (b) obesity defined as body mass index (BMI) ≥30,^43^ (c) presence of FM diagnosis provided by a rheumatologist according to the American College of Rheumatology (ACR) 2016 criteria; and (d) being able to read and sign an informed consent form. Patients were excluded according to the following criteria (a) presence of psychotic disorders or personality disorders; (b) ongoing or prior psychological treatment targeting FM management; and (c) having acute pain comorbidities or previously diagnosed chronic pain comorbidities distinct from FM.

Individuals who expressed a willingness to participate were invited to partake in an interview, with the rheumatologist who collected information on medical history, specifically the presence of other diagnoses of chronic pain disorders and prescription opioid use. Then they were referred to a licensed clinical psychologist for a second interview during which participants received information about the study, and they were given the opportunity to seek clarification on any queries or express any concerns. In addition, information regarding the patient’s medical history, including the presence of established diagnosis of personality disorders, psychotic disorders and previous or current psychological treatment was gathered. Also, each participant underwent The Structured Clinical Interview for DSM-5 Personality Disorders (SCID-5-PD) and the Psychotic Disorders module of The Structured Clinical Interview for DSM-5 (SCID-5). This assessment was conducted with the primary aim of excluding the presence of any previously undetected disorders.

After written informed consent was obtained, eligible participants were consecutively enrolled in the study. Data collection was conducted during the first week of the diagnostic assessment, before starting any rehabilitative program. A researcher (G.V.) supervised the completion of the questionnaires to ensure that all questions were answered.

## Measures

### Outcome

SI was assessed according to the individual answer at the item 9 of the Beck Depression Inventory-II (BDI-II)^44^ in its Italian validation.^45^ Item 9 is scored as follows: 0 (“I don’t have any thoughts of harming myself”), which indicates the absence of SI; 1 (“I have thoughts of harming myself, but I would not carry them out”), which indicate passive SI; 2 (“I would like to kill myself”); or 3 (“I would kill myself if I had the chance”), which indicate active SI. Item responses were dichotomized into: (*i)* presence of SI (assigned category of 1) in which we merged participants who scored 1, 2 and 3, and (*ii)* absence of SI (assigned category of 0) for those who reported a score of 0. This method is widely used in research to assess the presence of SI^32^^,^  ^46–48^ and has shown to have predictive validity for death by suicide and suicide attempts.^49^

### Predictors

Information on age (in years), BMI (BMI; calculated as kg/m^2^ where kg is a person’s weight in kilograms and m^2^ is their height in meters squared), pain duration (in years), presence of previous suicidal attempts (0 = no; 1 = yes) and current opioid use (0 = no; 1 = yes) was collected through a self-report form.

#### Pain intensity

The Numeric Pain Rating Scale (NPRS)^50^ was used to assess current perceived pain intensity. The NPRS consists of an 11-point scale (anchors 0 = “no pain”; 10 = “worst possible pain”).

#### Sleep quality

The Pittsburgh Sleep Quality Index^51^ is a 19-item self-reported questionnaire, which was used to assess sleep quality during the last month. Total score ranging from 0 to 21 with higher total score (ie, global score) indicating worse sleep quality. A global score greater than 5 is indicative of poor sleep quality. We used the Italian validation^52^ developed by Curcio et al. In the current study, internal consistency was good (Cronbach’s α = 0.82).

#### Depressive symptomatology

Levels of depressive symptomatology were evaluated with the Italian short-form version^53^ of the DASS-21,^54^ which consists of 3 subscales measuring anxiety, depression, and stress. For the purpose of this study, we used the depression subscale (DASS-D), which includes items that assess dysphoria, anhedonia, lack of incentive, and low self-esteem. This subscale consists of 7 items measured on a 4-point Likert scale ranging from 0 to 3. Total score ranges between 0 and 21. Higher scores indicate greater level of depressive symptoms (0–4: Normal; 5–6: Mild; 7–10: Moderate; 11–13: Severe; +14: Extremely severe). In the current study, internal consistency was good (Cronbach’s α = 0.80).

#### Pain catastrophizing

The Pain Catastrophizing Scale (PCS)^41^ is a self-report scale that evaluate pain-related catastrophic thinking. It consists of 13 items rated on a five-point Likert scale (from 0 = “not at all” to 4 = “all the time”), with total score ranging between 0 and 52. Higher scores indicates higher levels of pain catastrophizing. We used the Italian version^55^ of PCS which has good psychometric properties. In the current study, internal consistency was good (Cronbach’s α = 0.88).

### Statistical analysis

Data are described as means, standard deviations, and ranges for quantitative variables, and as frequencies and percentages for categorical variables. Normality was checked through visual inspection of normal Q–Q plots. The prevalence of SI was calculated using 95% CI proportion.

A preliminary set of univariate analyses was conducted to compare variables between individuals with and without SI, categorized based on item 9 of the Beck Depression Inventory (BDI) with the goal of identifying the variables of interest to be included in the next step. To classify patients into 2 groups based on the presence or absence of SI, the following procedure was implemented. Item responses were dichotomized as follows: (i) presence of SI (assigned category of 1), in which participants who scored 1, 2, and 3 were merged, and (ii) absence of SI (assigned category of 0), for those who reported a score of 0. Continuous variables from the initial sociodemographic form (ie, age, BMI, and pain duration) and the level of pain intensity, pain catastrophizing, sleep quality, and depression according to the scores reported at the relative questionnaire were compared in the 2 groups (individuals with SI vs those without SI) using independent *t*-tests. The effect size for the intergroup difference was calculated using Cohen’s d which was interpreted using the following benchmarks: Small (0.2), medium (0.5), and large (0.8). The level of significance was adjusted for multiple comparisons with Bonferroni correction (ie, α  ≤  0.05/7= *P *≤* *.007). On the other hand, categorical variables (ie, presence of previous suicide attempt, current opioid use) were compared using the Fisher exact test. Variables that showed statistically significant differences in the univariate analyses were then included in a multiple logistic regression model to identify factors independently associated with SI, while controlling for shared variance. All statistical analyses were performed using SPSS version 27.

## Results

One-hundred and sixty-nine individuals were assessed for eligibility. Thirteen were excluded for the following reasons: (i) 9 refused to participate, (ii) 4 met the exclusion criteria, specifically 1 individual reported a previous estrablished diagnosis of borderline personality disorder and 3 individuals a previous estrablished diagnosis of chronic migraine. Thus, 156 female participants were enrolled in the study. No missing data were present. Descriptive demographic and clinical characteristics of the sample are summarized in Table 1. According to the responses to item 9 of the BDI, 37.8% of participants reported presence of SI (95% confidence interval [CI]=30.2%–45.9%).

**Table 1.** Demographic and clinical characteristics of the total sample (*N* = 156)

	*n* (%)	Mean±SD	Range
Age in years		43.56±7.16	32–56
Body mass index		44.20±7.37	39–59
Pain duration in years		6.99±2.71	3–12
Presence of previous suicide attempt	2 (1.3%)		
Current opioid use	21 (13.5%)		
Presence of sucidal ideation	59 (37.8%)		
Pain Intensity		5.69±1.60	3–9
Pain catastrophizing		26.56±10.64	5–43
Depressive symptomatology		13.18±4.39	5–21
Sleep quality		10.92±5.07	2–19

## Univariate analyses

There was no statistically significant association between current SI and previous suicide attempts, as assessed using Fisher exact test (*P* = .527, 2-tailed). Only 2 participants reported previous suicide attempts, and they did not report current SI. However, we found a statistically significant association between current opioid use and the presence of SI (*P* = .027, 2-tailed). Among the 21 participants who reported current opioid use, 13 (61.9%) also reported the presence of SI.

Furthermore, our independent *t*-tests, as shown in Table 2, revealed significantly higher levels of pain intensity, pain catastrophizing, depressive symptomatology, and worse sleep quality in patients with SI compared to those without SI. Regarding pain catastrophizing, the observed effect size was large, while for pain intensity and depressive symptomatology, it was medium. Finally, the effect size for sleep quality was found to be small. Subsequently, these variables were entered into a multiple logistic regression model to assess their combined effects.

**Table 2.** Comparison of clinical, pain-related and psychological variables between patients with and without suicidal ideation.

	Abscence of suicidal ideation	Presence of suicidal ideation	*t*(154)	*P*	Cohen’s *d*
	*N* = 97	*N* = 59			
	Mean±SD	Mean±SD			
Age	42.75±6.92	44.90±7.42	−1.83	.70	.30
Pain duration	6.66±6.66	7.53±2.73	−1.96	.52	.32
BMI	43.48±7.85	45.71±5.42	−1.56	.121	.26
Pain intensity	5.25±1.53	6.41±1.45	−4.69	<.001*	.78
Pain catastrophizing	21.23±8.73	35.34±7.11	−10.48	<.001*	1.73
Depressive symptomatology	12.14±4.03	14.90±4.47	−3.78	<.001*	.655
Sleep quality	8.54±4.75	14.83±2.52	−9.40	<.001*	.155

Significant values (*P* value < .05) are in bold and indicate as *.

## Logistic regression model

A logistic regression was performed to ascertain the effects of current opioid use, pain intensity, sleep quality, depression, and pain catastrophizing on the likelihood that participants might display SI (0= absence of SI; 1 = presence of SI). The multiple logistic regression resulted in a significant final model (χ^2^(5) = 99.90, *P* < .001) with Nagelkerke *R*^2^ value of .64. The final model correctly classified 85.9% of cases; sensitivity was 88.1% and specificity was 84.5%. Notably, among the 5 predictors, only depressive symptomatology, sleep quality, and pain catastrophizing emerged as statistically significant factors (see Table 3). Specifically, higher levels of depressive symptomatology, pain catastrophizing, and sleep quality were associated with an elevated likelihood of experiencing SI.

**Table 3.** Logistic regression predicting likelihood of suicidal ideation based on opioid use, pain intensity, pain catastrophizing, depressive symptomatology, and sleep quality.

	B	SE	Wald	*df*	*P*	OR	95% CI for OR	
							Lower	Upper
Current opioid use	1.222	0.666	3.367	1	.067	3.395	0.920	12.527
Pain intensity	−0.116	0.183	0.400	1	.527	0.891	0.622	1.275
Pain catastrophizing	0.138	0.039	12.521	1	<.001*	1.148	1.063	1.239
Depressive symptomatology	0.181	0.058	9.753	1	.002*	1.198	1.070	1.342
Sleep quality	0.205	0.094	4.721	1	.030*	1.227	1.020	1.477

Signifcant values (*P* value < .05) are in bold and indicate as *.

## Discussion

Although numerous studies have investigated the factors associated with SI in individuals with chronic pain, there are few studies on FM, and none specifically on FM female patients with obesity. Thus, the purposes of this study were to (i) assess the prevalence of SI in female patients with FM and obesity, and (ii) to evaluate clinical, pain-related, and psychological factors associated with SI.

In our sample, the prevalence of SI was found to be 37.8%, which exceeds the lifetime SI prevalence of 20% found by Tang and Crane,^7^ as well as the point prevalence of 24% found by Racine et al.^56^ in mixed chronic pain patients. Trinanes et al., in a sample of Spanish female patients with FM (*N* = 117), identified a lower prevalence of 32.5%.^32^ Furthermore, our findings also indicate a higher SI prevalence than the overall SI prevalence of 29.57% reported in a recent meta-analysis on patients with FM.^57^ Trinanes et al. in their study, excluded individuals with comorbid chronic pain disorders that could potentially account for the reported pain symptoms. However, they did not provide specific information regarding exclusion criteria for psychopathology. In contrast, in our study participants with documented psychiatric disorders and personality disorders were excluded. Consequently, our findings primarily pertain to individuals without severe psychopathology, thereby increasing the worrisome nature of our results. Nevertheless, this discrepancy in the prevalence of SI in female patients with FM could be also attributed to the presence of obesity as a comorbid condition in our sample. Notably, obesity is associated with increased SI rates, especially among females.^58^^,^^59^ On the contrary, Calandre et al. reported a higher SI prevalence of 48% in their study involving 373 FM patients (including 20 men). However, it is important to note that Calandre et al. sample consisted of FM patients who exhibited more severe symptoms and/or were unresponsive to previous treatments, which may have contributed to the higher SI prevalence observed.

According to our results, depressive symptomatology, sleep quality and pain catastrophizing were significantly associated with the presence of SI. Depressive symptomatology emerges as one of the most consistent predictors of SI, both in the general population and among individuals with chronic pain.^7^^,^  ^48^^,^  ^60^ Regarding FM, 2 studies yielded results that align with our findings. The first by Trinanes et al.^32^ found significantly higher levels of depression (as measured by the BDI) in individuals affected by FM with SI compared to those without SI. Similarly, the second study conducted by Calandre and colleagues^47^ highlighted those individuals with FM and SI reported significantly higher BDI scores than FM patients without SI. Although our study employed different measures of depressive symptomatology (ie, DASS-D), our findings provide support to these previous results regarding the role of depressive symptomatology and its association with SI, while also considering other significant factors such as sleep quality and pain intensity. In addition, other factors commonly associated with depressive symptomatology (and not considered in our study) may contribute to SI in individuals with FM. For example, Ordóñez-Carrasco and colleagues^61^ identified 2 clusters of FM patients based on their vulnerability to SI in their cross-sectional study. They found that FM patients with high levels of perceived burdensomeness, thwarted belongingness, defeat, entrapment, psychological pain, and hopelessness face the highest risk of developing SI. Importantly, these factors have consistently been observed in the literature as being associated with depression.^62–64^

Sleep quality have emerged as a crucial risk factor for SI and suicide, with evidence indicating its significance independently of depression^65–67^ as observed in our current study. This relationship holds not only in the general popoulation^68^ but also among individuals with chronic pain.^56^ Moreover, it is more pronounced in women, particularly those with psychiatric and somatic comorbidities.^66^^,^^69^ Remarkably, recent research even suggests that sleep disturbance may play a mediating role in the link between chronic pain and SI.^70^ However, in patients with FM evidence is scarce. Available studies indicate that poor sleep quality is independently associated with SI in patients with migraine and comorbid FM.^71^ Consistent with this, Calandre et al.,^47^ found significantly poorer sleep quality in FM patients with SI compared to those without SI. Sleep disturbance is hypothesized to contribute to increased SI by impairing decision-making ability, impulse control, and emotion regulation.^69^^,^^72^ Various factors have been proposed as mediators in the relationship between sleep and suicide, as discussed by Woznica and colleagues.^69^ Among these factors, serotonergic dysregulation has received significant attention as a biological mediator linking sleep and suicide.^73^ Mood dysregulation may also play a role, as disruptions in sleep-mood regulation processes and subsequent disturbances in emotional reactivity can contribute to cognitive and physiological arousal that perpetuates sleep problems. Additionally, cognitive deficits resulting from sleep deprivation may promote impulsivity, serving as additional mediators in the association between sleep and suicide.

In the present study, pain catastrophizing emerged as another significant factor associated with SI. Although there is no specific evidence on FM, our results align with a study conducted by Edwards and colleagues,^33^ in which pain catastrophizing independently predicted both the presence and severity of SI in patients with chronic pain, even after controlling for depression and anxiety. Pain catastrophizing involves an exaggerated and negative cognitive appraisal of pain, often accompanied by a sense of helplessness and a belief that the situation is unbearable or unsolvable. By amplifying the perceived threat and emotional distress, catastrophizing may contribute to an increased risk of SI. There is one dimension of pain catastrophizing that we think deserves considerable attention in the future in relation to SI, namely, rumination. The ruminative component of pain catastrophizing involves repetitive, intrusive, negative thinking, and difficulty disengaging. Importantly, according to a systematic review, rumination is associated to both SI and suicidal behavior.^74^ Longitudinal studies have yielded empirical evidence establishing temporal precedence in the interplay between rumination and SI, thus implying that rumination might serve as a prospective risk factor in the genesis of SI.^75^ However, no evidence are available on the role of rumination in chronic pain. Our results underscore the significant role of catastrophizing in predicting SI, above and beyond the contribution of depression and sleep quality and contribute to a more nuanced understanding of the complex factors involved in the development of SI.

Obesity poses a significant challenge in this context, given its well-established links to both depression ^76^^,^^77^ and sleep quality.^78^^,^^79^ However, the relationship between pain catastrophizing and obesity in patients with chronic pain remains poorly understood. A study on patients with osteoarthritis and comorbid severe obesity reports higher levels of catastrophizing compared to their counterparts with obesity and overweight.^80^ Even though these results cannot be generalized to the population of FM patients, they potentially indicate that obesity may exert an adverse influence on this factor as well.

Interestingly, although patients with SI exhibited higher pain intensity in the univariate analyses, this association did not retain significance within the logistic regression model when considering other variables. This finding suggests that, concerning SI, psychological factors and sleep quality may hold greater significance than pain intensity per se. Our results are partially in line with the existing literature. Indeed, the role of pain intensity is still debated, and evidences are sparse and contradictory. Overall, as reviewed by Racine,^81^ most studies on the pain-suicide relationship in chronic pain patients have failed to establish pain intensity as a significant risk factor for suicidality. A study comparing individuals with FM to those with low back pain found a higher risk of SI in the FM group, despite comparable pain intensity levels.^30^ Conversely, other evidence has reported an association between SI and pain intensity.^31^ At this stage the role of the pain intensity in suicidality should be further investigated. Nonetheless, clinicians involved in suicide prevention for FM should not disregard the potential impact of pain intensity. However, if it is the psychological response to pain, rather than pain itself, that exerts influence on SI, it becomes imperative to carefully account for the individual psychological interpretation of the experience of pain in individual assessments.

Finally, participants with SI did not report higher levels of BMI. Our finding conflicts with other evidence higlighting an association between higher BMI and increased risk of SI and suicidal behaviors.^82^^,^^83^ However, the available evidence is mixed; in fact, other studies have not found an association between BMI and SI.^84^ One explanation could be that there is not a linear association between BMI and SI, as suggested by the study conducted by Dutton et al.,^58^ but that this relationship may be mediated by other constructs such as perceived burdensomeness, a factor not assessed in our study.

In conclusion, an assessment of SI should be an essential part of the evaluation of female individuals suffering of FM associated with obesity. Psychological factors, including pain catastrophizing and depression, as well as sleep-related components, should be considered. Indeed, our study highlights the importance of considering multiple risk factors in suicide risk assessment. While depressive symptomatology and sleep quality are recognized indicators of SI, our results emphasize that pain catastrophizing provides unique and valuable information in identifying individuals who may be at heightened risk for SI. By integrating measures of pain catastrophizing into comprehensive suicide risk assessments, clinicians can better identify individuals who may benefit from targeted interventions aimed at reducing pain catastrophizing and preventing the escalation of SI. Importantly, pain catastrophizing can be effectively addressed through cognitive-behavioral therapy (CBT)^35^ and is, in fact, a primary target of CBT for chronic pain. By addressing the cognitive and emotional aspects underlying pain catastrophizing, CBT can help individuals develop a more adaptive appraisal of their pain experiences.

It is important to acknowledge the limitations of our study. The sample consisted exclusively of female patients with FM and obesity receiving treatment at a tertiary rehabilitation unit, which may not be representative of the entire FM population. Nevertheless, given that 35.7% of FM patients have obesity, these individuals represent a substantial proportion of the FM spectrum.^25^ The assessment of SI based on a single item (in this case the item #9 of the BDI-II) is suboptimal but efficient. While this method has been used in various studies,^32^^,^  ^46^^,^^47^^,^  ^85^ a more comprehensive assessment using clinical interviews or self-report measure that evaluate specifically SI is recommended. In light of our study's exclusion criteria, which precluded the participation of individuals with previous established diagnoses chronic pain conditions other than FM, it is important to acknowledge the potential presence of these conditions, albeit undiagnosed. Finally, due to the cross-sectional design of our study, we were only able to examine the association between depressive symptomatology, sleep quality, pain catastrophizing, and SI, without establishing a causal relationship. To overcome this limitation and corroborate our findings, future research should employ longitudinal designs.

## Conclusion

In summary, this study found a high prevalence of SI among women with FM and obesity. The presence of SI was associated with depressive symptomatology, sleep quality, and pain catastrophizing. These results suggest that it is not merely the intensity of perceived pain that contributes to SI, but rather the complex interplay between pain related, psychopathological and sleep-related factors. These findings highlight the need to develop and evaluate targeted interventions aimed at addressing these factors to prevent and reduce SI in this vulnerable population. Psychological interventions focusing on pain management should incorporate the treatment of depressive symptoms, sleep quality, and pain catastrophizing as potential areas of intervention. Future research should investigate the efficacy of such interventions in reducing SI in individuals with FM and comorbid obesity.
