# Corrigendum to: Suicidal ideation in female individuals with fibromyalgia and comorbid obesity: prevalence and association with clinical, pain-related, and psychological factors

**DOI:** 10.1093/pm/pnae037

**Published:** 2024-05-11

This is a corrigendum to: Giorgia Varallo, Federica Scarpina, Tor Arnison, Emanuele Maria Giusti, Micheal Tenti, Giada Rapelli, Roberto Cattivelli, Giulia Landi, Eliana Tossani, Silvana Grandi, Christian Franceschini, Valentina Baldini, Giuseppe Plazzi, Paolo Capodaglio, Gianluca Castelnuovo, Suicidal ideation in female individuals with fibromyalgia and comorbid obesity: prevalence and association with clinical, pain-related, and psychological factors, *Pain Medicine*, Volume 25, Issue 3, March 2024, Pages 239–247, https://doi.org/10.1093/pm/pnad139

The originally published version of this article has been corrected.

The Funding statement has been updated to read as follows:

This work was supported by Italian Ministry of Health - Ricerca Corrente.

